# Molecular optimization using a conditional transformer for reaction-aware compound exploration with reinforcement learning

**DOI:** 10.1038/s42004-025-01437-x

**Published:** 2025-02-08

**Authors:** Shogo Nakamura, Nobuaki Yasuo, Masakazu Sekijima

**Affiliations:** 1 Department of Life Science and Technology, Institute of Science Tokyo, 4259–J3–23, Nagatsuta-cho, Midori-ku, Yokohama, 226-8501 Kanagawa Japan; 2 Academy for Convergence of Materials and Informatics (TAC-MI), Institute of Science Tokyo, S6-23, Ookayama, Meguro-ku, 152-8550 Tokyo Japan; 3 Department Computer Science, Institute of Science Tokyo, 4259–J3–23, Nagatsuta-cho, Midori-ku, Yokohama, 226-8501 Kanagawa Japan

**Keywords:** Cheminformatics, Computational chemistry, Structure prediction, Drug discovery and development

## Abstract

Designing molecules with desirable properties is a critical endeavor in drug discovery. Because of recent advances in deep learning, molecular generative models have been developed. However, the existing compound exploration models often disregard the important issue of ensuring the feasibility of organic synthesis. To address this issue, we propose TRACER, which is a framework that integrates the optimization of molecular property optimization with synthetic pathway generation. The model can predict the product derived from a given reactant via a conditional transformer under the constraints of a reaction type. The molecular optimization results of an activity prediction model targeting DRD2, AKT1, and CXCR4 revealed that TRACER effectively generated compounds with high scores. The transformer model, which recognizes the entire structures, captures the complexity of the organic synthesis and enables its navigation in a vast chemical space while considering real-world reactivity constraints.

## Abstract (web summary)

Designing molecules with desirable properties is a critical endeavor in drug discovery, however, synthetic accessibility remains limited in the existing compound exploration models. Here, the authors report TRACER as a framework that integrates molecular property optimization with synthetic pathway generation, generating compounds targeting DRD2, AKT1 and CXCR4 with high reward values.

## Introduction

Small molecules constitute one of the essential modalities in drug discovery. However, new drug candidates have become increasingly difficult to discover, and some take more than 12 years to develop and cost an average of $2.6 billion to bring to the market^1,2^. In the early stages of research, hit compounds with high inhibitory activity against the target protein are often identified via high-throughput screening or virtual screening. These hit compounds are then structurally expanded by adding building blocks to explore compounds with the desired properties^3^. To find drug candidate compounds within a vast chemical space by starting from hit compounds,improving the efficiency of the DMTA cycle, which is a process used in drug discovery to design, make, test, and analyze compounds, is crucial.

Recent advances in deep learning models and their generalization capabilities have led to the development of generative models for de novo design and hit-to-lead optimization purposes^4^. However, most of these methods focus only on “what to make” and do not sufficiently consider “how to make”. Molecular generative models utilizing various architectures such as recurrent neural networks (RNNs)^5–8^, variational autoencoders (VAEs)^9,10^, and generative adversarial networks (GANs)^11,12^, as well as molecular design methods that explicitly consider interactions with target proteins^13–15^ have been developed. Since these methods do not consider how to synthesize the generated molecules, chemists must search for candidates for synthetic experiments from the numerous compounds suggested by the employed generative models; this aspect is a significant barrier preventing a shift to bench synthesis^16^.

The most commonly used scoring function for estimating synthetic ease, the SA score^17^, penalizes fragments, chiral centers, and certain substructures that are rarely seen in the PubChem database. Recently, Chen et al. developed a scoring method using building blocks and chemical reaction datasets to improve the SA score^18^. However, these topological methods are well suited for providing a rough estimate of the ease of synthesis in a high-throughput manner and cannot truly consider synthetic feasibility^19^. Although retrosynthesis models have been proposed to computationally predict synthetic pathways^20^, they are unable to consider the complexity of chemical reactions; thus, the knowledge and experience of human experts are indispensable during the synthesis planning process^21^. Despite the recent advances in machine learning-based scoring functions^22–27^, these functions predict only the results of retrosynthesis models. Therefore, the fundamental problems of these models remain unsolved^21^.

**Fig. 1:**
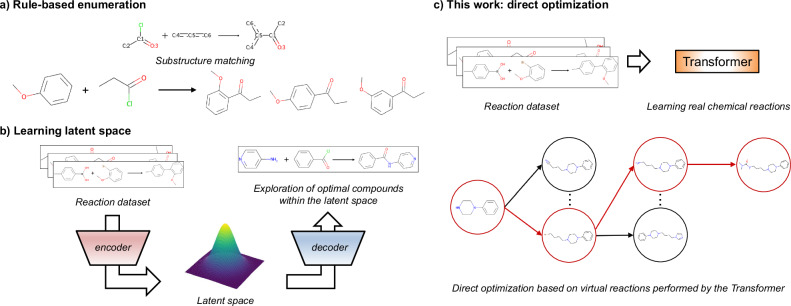
Comparison of prior studies and the proposed method. **a** Scheme of a rule-based methods. In this example, the ortho-para orientation and steric hindrance are disregarded. **b** Outline of the previous works. This type of model, which learns a latent space representation, enables smooth compound exploration within the learned distribution. **c** Our work. A direct tree search occurs from the starting material on the basis of the virtual reactions performed by a conditional transformer model trained on real chemical reactions.

One approach for addressing the problem of generating synthesizable molecules is to construct virtual libraries through molecular design tasks with synthetic pathways by using predefined sets of reaction templates, which describe how reactants can be transformed into products^28–34^. Advances in machine learning have led to the development of deep learning-based analogous methods in combination with optimization algorithms (e.g., genetic algorithms or Bayesian optimization)^35–38^. These methods generate molecules that are similar to a given input compound in a descriptor space, such as extended connectivity fingerprints (ECFPs), and they also generate reaction pathways described by reaction templates. Reinforcement learning models, such as actor-critic and Monte Carlo tree search (MCTS), have been reported to be able to generate optimized compounds^39–42^ by training neural networks to predict reaction templates and building blocks that maximize the value of the reward function. Recently, a template-based method utilizing GFlowNet was reported for generating compounds with a probability that is proportional to a reward value^43,44^. However, the reaction templates used in these models are oversimplified, and chemoselectivity and regioselectivity, which are important aspects in real chemical reactions, are challenging to consider (Fig. 1a). Even though these selectivities may be considered by describing the patterns of the target reaction in detail, the scope of the substrates for all possible reactions is unrealistic to comprehensively define. Since new chemical reactions are reported daily, describing detailed chemical transformations is a time-consuming process.

As a data-driven approach, the development of structural optimization methods that can explicitly learn chemical reactions has the potential to address the above challenges. Bradshaw et al. proposed models that embed molecules or reaction trees into a latent space, which is a low-dimensional representation learned from the input data; here, similar data points (i.e., chemical structures or reaction trees) are embedded to make them close together^45,46^. The Molecule Chef^45^ learns the latent space from a “bag of reactants” (summation of reactant embeddings) and connects these reactants to the property of the product predicted by Molecular Transformer^47^. Despite the fact that this process enables compound generation while considering real chemical reactions, it can only handle single-step reactions. DoG-Gen^46^ has succeeded in mapping a latent vector of reaction trees via a hierarchical gated graph neural network (GGNN), achieving multistep synthetic pathway generation. However, in structural optimization cases, DoG-Gen cannot follow the drug discovery process; it is incapable of generating compounds by starting from specific hit compounds.

In this study, we propose TRACER, which is a molecular generation model that combines a conditional transformer and an MCTS. The transformer model is constructed on the basis of attention mechanism, which allows it to selectively and directly focus on the important components contained in the input sequence data^48^. Therefore, the transformer model trained on chemical reactions such as the structural transformation contained in the simplified molecular input line entry system (SMILES)^49^ is expected to recognize the substructures that affect reactions from the whole reactants, achieving high accuracy in forward synthesis prediction tasks^47^. Previously developed data-driven methods^45,46^ generate optimized structures in the latent space, so they can search for compounds with the desired properties in a structure that smoothly fills a continuous space (Fig. 1b). In contrast with previous works that used end-to-end architectures, our proposed framework decouples the transformer as a product prediction model from the MCTS, which is used as a structural optimization algorithm. Therefore, TRACER enables direct optimization on the basis of virtual chemical reactions; thus, it is expected to propose structures and synthetic pathways that are far from the training dataset (Fig. 1c). Our model learns 1000 reaction types that are associated with a real chemical conversions, enabling the model to learn more fine-grained information than that of the rule-based methods, which use approximately 100 reaction templates^35,36,38–42^. With the continuous growth of chemical reaction databases, the model needs to be applied to an increasingly diverse range of reactions. However, transformer models have been verified to follow scaling laws^50^ in natural language processing tasks, indicating that increasing the number of model parameters can effectively address this challenge.

## Results and discussion

### Effect of the conditional token on chemical reactions

**Fig. 2:**
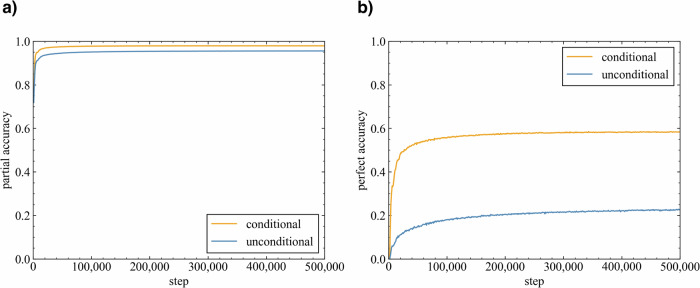
Evaluations of the conditional transformer (orange) and unconditional transformer (blue). **a** Partial accuracy (calculated per SMILES token). **b** Perfect accuracy (calculated per molecule).

In this study, the transformer model was trained on molecular pairs created from chemical reactions via the SMILES sequences of reactants and products as source and target molecules, respectively. Following the evaluation method proposed by Yoshikai et al.^51^, partial accuracy (the accuracy per SMILES token) and perfect accuracy (the accuracy per molecule) were calculated for the validation data to assess the ability of the model to recognize partial and whole product structures. The impact of including or excluding reaction template information on the accuracy of the model was investigated (Fig. 2). The partial accuracy quickly reached approximately 0.9, regardless of the presence or absence of the reaction template information; this result indicated the ability of the model to learn partial product structures from the reactants, which aligned with the chemical knowledge that the scaffolds of reactants often remain in the products. However, the perfect accuracy increased more slowly than the partial accuracy did in both the unconditional and conditional models; thus, learning the entire product structure required more time. The perfect accuracy plateaued at approximately 0.6 for the conditional model and 0.2 for the unconditional model. These results indicated that the model had difficulty reproducing the chemical reactions from the training data without the reaction templates, and the accuracy improved when this information was provided. These results indicate the fact that a single substrate can undergo numerous chemical reactions and yield various products in the absence of additional constraints.

The top-n accuracies attained by the models for the test data were calculated (Table 1). The top-1, top-5, and top-10 accuracies were improved by adding information to the reaction templates. These results indicate that knowledge regarding the chemical reactions narrows the chemical space for predicting molecules and improves the ability of the model to generate appropriate products from the learned chemical reactions.

**Table 1:** The top-n accuracies achieved by the transformer models

Model	Top-1 (%)	Top-5 (%)	Top-10 (%)
Conditional	55.1	67.2	71.7
Unconditional	26.6	42.5	47.2

**Fig. 3:**
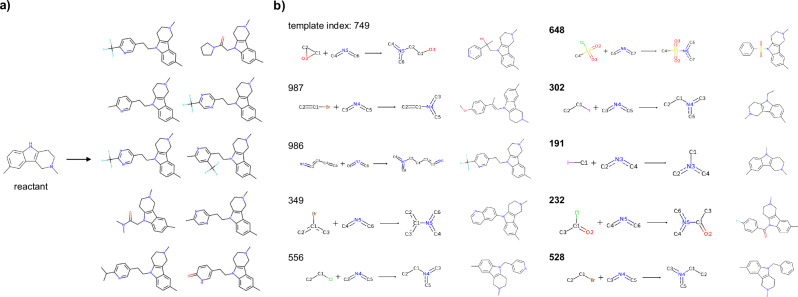
Examples of the generated molecules. **a** Examples generated by the unconditional transformer. The top 10 compounds for which the beam width was set to 100 are shown. **b** Compounds generated by the conditional transformer. The numbers indicate the indices of the reaction templates predicted by the GCN. The bolded indices represent novel compound-reaction template combinations that did not exist in the training data. The generated molecules were the top-1 results when the beam width was set to 10.

In some cases, bias was observed in the products generated by the unconditional transformer from the reactants. Figure 3a shows an example where the top-10 products were generated by a beam search with a beam width of 100 in the trained unconditional transformer, where the SMILES sequence of the depicted reactant was used as the input. The generated products were biased toward compounds transformed by reactions involving Csp(3) bonds to an indole-type nitrogen atom; these results were attributed to the bias in the chemical reactions involving similar transformations in the USPTO dataset. In contrast, by using the conditional transformer and the reaction templates predicted by the GCN, compounds could be generated through diverse chemical reactions (Fig. 3b). The GCN selected not only combinations of the reactants and reaction templates included in the training data, such as epoxide ring opening reactions and Buchwald-Hartwig cross-coupling with vinyl halides but also combinations of compounds and reaction templates that were not present in the training data, such as amidation and sulfonamidation, and successfully generated reasonable product candidates. These results indicated that the conditional transformer can extract chemical reaction knowledge from conditional tokens and propose rational products even for unknown combinations of reactants and chemical reactions. An example of the product generation results produced under the condition of unmatched reaction templates, where no partial structure matches were found, is presented in Supplementary Fig. 1.

### Optimization of compounds involving the generation of synthetic routes targeting specific proteins via a QSAR model

**Fig. 4:**
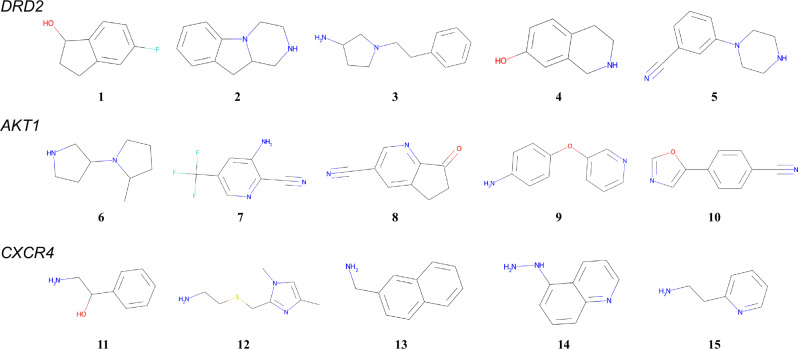
The set of molecules selected as starting materials. For each target protein, QSAR values were calculated for preprocessed compounds. Starting compounds were then determined by randomly sampling one compound from each QSAR value bin: 0-0.1, 0.1-0.2, 0.2-0.3, 0.3-0.4, and 0.4-0.5.

Five starting materials were selected for DRD2, AKT1, and CXCR4 from the USPTO 1k TPL dataset. The detailed procedure is described in Section 4.5, and the selected molecules are shown in Fig. 4. Utilizing these reactants as the root nodes, the MCTS calculations from selection to backpropagation were executed for 200 steps. The number of reaction templates predicted in the expansion step was set to 10. These hyperparameters could be adjusted on the basis of the available computational resources.

**Fig. 5:**
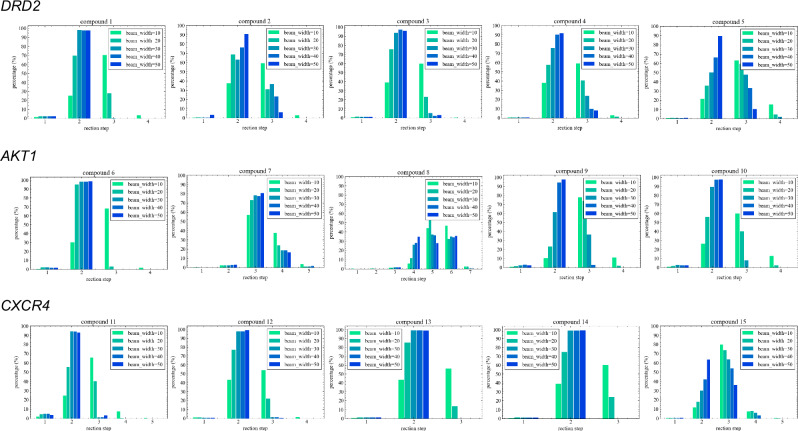
Distribution of the reaction steps. The figure shows the distribution of reaction step counts for generated compounds, obtained via the transformer with inference beam widths ranging from 10 to 50.

The compounds generated by the MCTS were evaluated in terms of the beam width in the transformer inference process, the total number of generated molecules (Total), the ratio of the number of unique molecules to those contained in the USPTO dataset, the number of molecules (Num) and the proportion of molecules with active prediction probabilities exceeding the threshold of 0.5 imposed by the QSAR model (Tables 2, 3, and 4). In addition to these metrics, to confirm the depth of the MCTS, the proportions of the reaction steps for the generated compounds were investigated (Fig. 5). A uniqueness analysis revealed that TRACER efficiently generated compounds that were distinct from those in the USPTO database across all target proteins. The efficiency of generating compounds with QSAR values exceeding 0.5 was significantly influenced by the starting compound. For DRD2 and AKT1, when the starting material had a high QSAR value, the structural exploration process started from scaffolds similar to those of the active compounds learned by the QSAR model, leading to a higher ratio of discovered molecules with high reward values. However, even when starting with compounds possessing low QSAR values, TRACER successfully generated compounds with high QSAR values by adjusting the beam width of the transformer. It was difficult to discover optimized compounds for CXCR4 when starting from compounds with low QSAR values (starting materials **11** and **12**). However, when starting from compounds **13-15**, our model successfully generated compounds with QSAR values exceeding 0.5 at a rate of approximately 15%. Starting from compounds **11,** **12**, and **15**, it was challenging to improve the QSAR value. This is likely because the number of known CXCR4 ligands is relatively small, and few scaffolds are similar to the active compounds learned by the QSAR model that can access from these starting materials, thus limiting the opportunities for further development.

**Table 2:** Structural optimization results obtained for DRD2

Starting Material (SM)	QSAR of the SM	Beam Width	Total	Uniqueness to USPTO (%)	QSAR > 0.5	
					Num	Diversity
**1**	0.08	10	5277	99.8	624	0.437
					(11.8%)	
		20	11107	99.7	1238	0.437
					(11.1%)	
		30	16577	99.9	**1984**	0.442
					(**12.0**%)	
		40	21119	99.9	1622	0.435
					(7.67%)	
		50	24339	99.9	795	0.396
					(3.26%)	
**2**	0.12	10	5451	100	**407**	0.405
					(**7.47**%)	
		20	7170	100	418	0.387
					(5.82%)	
		30	9956	100	128	0.364
					(1.28%)	
		40	13034	100	185	0.373
					(1.42%)	
		50	15040	100	255	0.370
					(1.69%)	
**3**	0.27	10	4232	99.4	**526**	0.348
					(**12.4%**)	
		20	8576	99.7	823	0.368
					(9.57%)	
		30	14297	99.9	1046	0.361
					(7.31%)	
		40	19926	99.8	987	0.395
					(4.95%)	
		50	25496	99.9	1708	0.382
					(6.69%)	
**4**	0.38	10	8992	99.9	843	0.416
					(9.36%)	
		20	23009	100	2790	0.413
					(12.1%)	
		30	37051	100	4371	0.417
					(11.8%)	
		40	50350	100	6017	0.427
					(11.9%)	
		50	64190	100	**8319**	0.425
					(**13.0**%)	
**5**	0.47	10	3092	99.9	618	0.369
					(20.0%)	
		20	4895	99.9	1618	0.349
					(33.0%)	
		30	7652	99.9	**3346**	0.342
					(**43.7**%)	
		40	8895	99.9	3815	0.330
					(42.8%)	
		50	11845	99.9	4538	0.348
					(38.3%)	

The highest efficiency achieved for each beam width is shown in bold.

**Table 3:** Structural optimization results obtained for AKT1

Starting Material (SM)	QSAR of the SM	Beam Width	Total	Uniqueness to USPTO (%)	QSAR > 0.5	
					Num	Diversity
**6**	0.07	10	5243	100	**713**	0.437
					(**13.6**%)	
		20	6526	100	488	0.413
					(7.47%)	
		30	12026	100	772	0.423
					(6.42%)	
		40	18985	100	1383	0.430
					(7.28%)	
		50	27355	100	2360	0.433
					(8.63%)	
**7**	0.16	10	5992	100	766	0.463
					(12.9%)	
		20	8573	100	1528	0.445
					(17.8%)	
		30	6650	100	**1414**	0.436
					(**21.3**%)	
		40	7275	100	925	0.367
					(12.7%)	
		50	10595	100	983	0.371
					(9.28%)	
**8**	0.23	10	2742	99.6	426	0.491
					(15.5%)	
		20	4383	99.7	**725**	0.474
					(**16.5**%)	
		30	5599	99.7	837	0.491
					(14.9%)	
		40	7425	99.8	1044	0.491
					(14.0%)	
		50	9085	99.8	1191	0.483
					(13.1%)	
**9**	0.31	10	3299	99.9	**1068**	0.406
					(**32.4**%)	
		20	5415	100	1230	0.413
					(22.7%)	
		30	6901	100	873	0.404
					(12.6%)	
		40	10017	100	867	0.375
					(8.65%)	
		50	12943	100	1155	0.378
					(8.92%)	
**10**	0.48	10	3557	99.7	1072	0.503
					(30.1%)	
		20	6690	99.9	2052	0.473
					(30.7%)	
		30	9554	99.9	3404	0.454
					(35.6%)	
		40	11666	99.9	4804	0.460
					(41.2%)	
		50	14478	99.9	**6504**	0.471
					(**44.9%**)	

The highest efficiency achieved for each beam width is shown in bold.

**Table 4:** Structural optimization results obtained for CXCR4

Starting Material (SM)	QSAR of the SM	Beam Width	Total	Uniqueness to USPTO (%)	QSAR > 0.5	
					Num	Diversity
**11**	0.07	10	7326	99.8	**230**	0.468
					(**3.13**%)	
		20	15999	99.9	387	0.491
					(2.41%)	
		30	24524	99.9	187	0.448
					(0.76%)	
		40	35599	99.9	301	0.447
					(0.84%)	
		50	45249	99.9	266	0.434
					(0.58%)	
**12**	0.12	10	2546	99.9	95	0.402
					(3.72%)	
		20	3705	99.9	**185**	0.334
					(**5.00**%)	
		30	4580	100	93	0.308
					(2.03%)	
		40	6617	100	197	0.313
					(2.98%)	
		50	9789	100	314	0.317
					(3.21%)	
**13**	0.22	10	6362	99.9	926	0.460
					(14.5%)	
		20	12631	99.9	1524	0.454
					(12.1%)	
		30	21124	99.9	2728	0.450
					(12.9%)	
		40	28208	100	4373	0.453
					(15.5%)	
		50	34727	100	**5821**	0.458
					(**16.8**%)	
**14**	0.32	10	6571	99.8	542	0.454
					(8.23%)	
		20	12596	99.9	1065	0.457
					(8.45%)	
		30	16343	99.9	1764	0.450
					(10.8%)	
		40	20708	99.9	**3203**	0.469
					(**15.5**%)	
		50	20464	99.9	2792	0.471
					(13.6%)	
**15**	0.43	10	4855	99.5	657	0.477
					(13.5%)	
		20	9558	99.7	**1395**	0.486
					(**14.6**%)	
		30	16378	99.8	915	0.475
					(5.58%)	
		40	25472	99.9	1460	0.484
					(5.72%)	
		50	35467	99.9	2284	0.487
					(6.43%)	

The highest efficiency achieved for each beam width is shown in bold.

Overall, the optimal beam width for the conditional transformer model was found to vary depending on the starting compound. As shown in Fig. 5, increasing the beam width led to a reduced depthwise exploration process. In contrast, a narrower beam width was observed to increase the depth, leading to a greater number of reaction steps. These results indicated that, in some cases, promoting depthwise exploration by narrowing the tree width was more effective for discovering molecules with high reward values than dispersing the nodes to be explored by widening the tree width (starting materials **2-3,** **6,** **9**, and **11**). Conversely, in other cases, increasing the beam width facilitated the generation of high-reward molecules by promoting breadth-wise exploration (starting materials **4,** **10**, and **13**). These findings demonstrate that TRACER is capable of performing structural optimization when starting from various initial compounds by optimizing the beam width.

To investigate the potential impact of increasing the beam width on the diversity of the generated molecules, the internal diversity of the compound sets that exceeded the threshold was calculated. In some cases, decreasing diversity trends were observed when the beam width was increased (starting materials **1,** **3,** **7,** **9**, and **12**), whereas no notable influences were observed for the other materials.

In our model, since some reactants are treated implicitly, the reactants that are newly added to the input compounds were also investigated. For all pathways generated in Tables 2, 3, and 4, a total of 20,765 diverse reactants were identified, enabling this model to generate molecules with unique structures relative to those of the USPTO dataset.

### Reaction pathways generated by the model

Figure 6 shows the compounds with the highest reward values and their synthesis routes for each of the starting materials. In these synthetic pathways, all reactants were not explicitly addressed. Therefore, the availability of the building blocks shown in these figures was investigated via Reaxys^52^, and all the starting materials were found to be commercially available (Supplementary Figs. 2–16). This result indicated that a wide range of reactants in USPTO are purchasable or synthesizable and that the conditional transformer captured this feature even though the model implicitly learned part of the reactants.

**Fig. 6:**
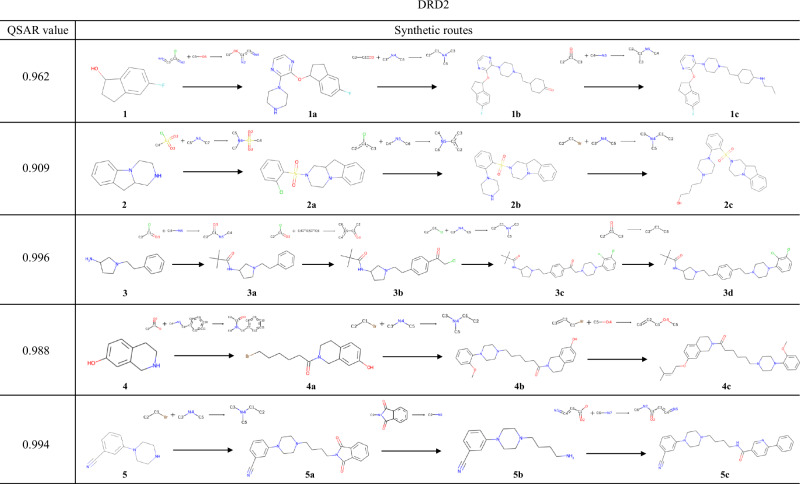
Compounds with the highest QSAR values for DRD2 and their synthesis routes. The compound with the highest reward value and its synthesis pathway, generated from experiments starting with compounds **1**–**5**, which were selected to target DRD2.

Starting from **1**, a series of reactions, including a nucleophilic attack of an alcohol on an aryl chloride and reductive amination were selected; this resulted in the generation of **1c** with a QSAR value of 0.962. During the synthesis of **1a**, the aryl fluoride scaffold of **1** and the newly added 2-chloropyrazine (Supplementary Fig. 2) could compete in an aromatic nucleophilic substitution reaction. However, several similar reactions are contained in the USPTO dataset, where aliphatic alcohols react with chloropyrazine, and an actual patents also exist^53^. This result indicates that TRACER correctly predicted the selectivity of the aromatic nucleophilic substitution reaction by learning real chemical reactions. Predicting such selectivity is extremely difficult with reaction template-based methods that determine applicable reaction templates without considering real chemical reactions. For compound **2**, sulfonamidation of the amine was utilized to synthesize **2a**, followed by conducting nucleophilic substitution and a nucleophilic attack on the alkyl halide to yield **2c**. In the case of **3,** **3a** was generated by amidation with pivaloyl chloride. **3a** was converted to **3b** via Friedel-Crafts acylation. Generally, when an *sp*^3^ carbon is bonded to an aromatic ring, Friedel-Crafts reactions proceed selectively at the para position^54^, which the conditional transformer model successfully predicted. The derivation from **4** involved the amidation and two subsequent nucleophilic attacks of heteroatoms on alkyl halides to produce **4c**. When **5** was used as the starting material, the introduction of the aliphatic amine moiety on the piperazine nitrogen using a phthalimide derivative provided **5b**, and subsequent amidation provided **5c** with a QSAR value of 0.994.

**Fig. 7:**
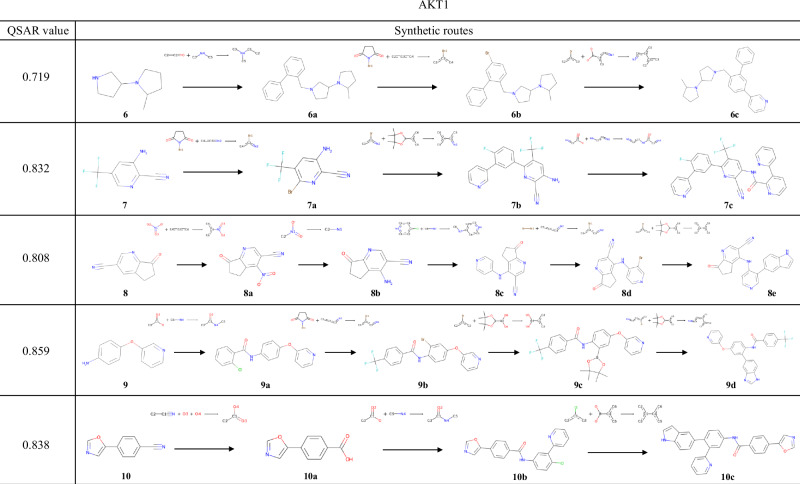
Compounds with the highest QSAR values for AKT1 and their synthesis routes. The compound with the highest reward value and its synthesis pathway, generated from experiments starting with compounds **6**–**10**, which were selected to target AKT1.

**Fig. 8:**
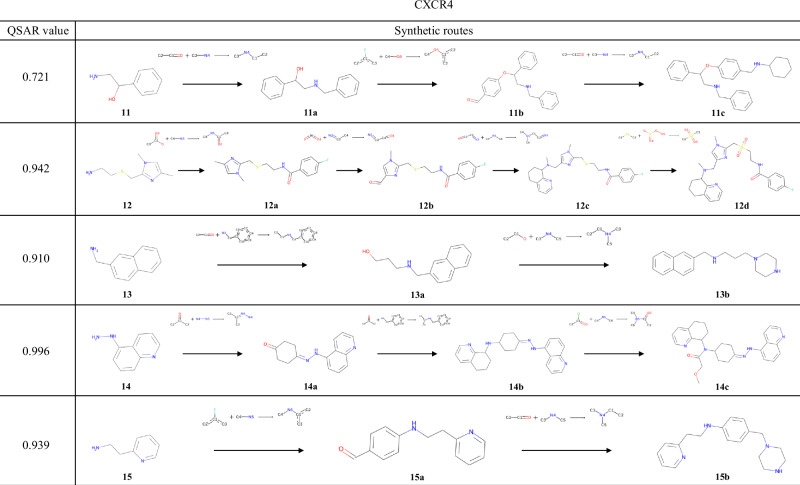
Compounds with the highest QSAR values for CXCR4 and their synthesis routes. The compound with the highest reward value and its synthesis pathway, generated from experiments starting with compounds **11**–**15**, which were selected to target CXCR4.

The results of the experiments conducted using the QSAR models of AKT1 and CXCR4 are shown in Figs. 7 and 8, respectively. Several examples were found in these reaction pathways, where the selectivities of the chemical reactions were correctly predicted. In the transformation from **8a** to **8b**, a method for reducing a nitro group while retaining a cyano group has been reported^55^. During the synthesis of **8d**, the substitution at the 3-position of pyridine and the ortho-para directing effect of an aniline were correctly predicted. In the synthesis from **9a** to **9b**, similar reactions in which the ortho position of the amide groups in the substrates is halogenated using a metal catalyst^56,57^ have been reported. For **11a**, the exact same reaction has been reported, in which reductive amination occurs while the alcohol is retained^58^. In the transformation from **11a** to **11b**, the aryl fluoride undergoes a substitution reaction while retaining the aldehyde of the newly added building block; indeed, several such examples exist^59,60^. As shown in these examples, TRACER can predict the selectivity of chemical reactions by explicitly learning actual chemical reactions.

Although the structural optimization process using an unconditional transformer was explored, the generated synthetic routes proved impractical due to the use of several unreasonable transformations, such as multiple-step reactions performed in a single inference, the insertion of carbon atoms into alkyl chains, and a disregard for selectivity. Therefore, the retention rate of the Murcko scaffold^61^ of the reactants was investigated for the routes generated via structural optimization, with the QSAR model of DRD2 as a reward function. The retention rate was 94.5% for the conditional transformer, whereas it was 43.3% for the unconditional transformer. Considering these results together with the perfect accuracy investigation conducting in Section 5.3, it can be concluded that conditioning on chemical reactions is crucial.

### Comparative analysis with molecular generation models that incorporate synthetic route generation

To evaluate TRACER, structural optimization experiments were conducted on the QSAR models of DRD2, AKT1, and CXCR4 along with benchmark models (the Molecule Chef^45^, DoG-Gen^46^, CasVAE^37^, and SynFlowNet^43^). A comparison among these models is presented in Table 5. The specification of starting compounds, which is a crucial function in drug discovery, is supported only by our framework. Furthermore, the models that are capable of explicitly learning chemical reactions are limited to TRACER, the Molecule Chef, and DoG-Gen. Notably, the Molecule Chef is applicable only to single-step reactions. Despite the fact that CasVAE and SynFlowNet, which are rule-based methods that utilize reaction templates, can perform structural optimization accompanied by the generation of reaction pathways, they are unable to consider actual chemical reactions. The optimization methods proposed by each model are described in the following section.

**Table 5:** Comparison among the features of the benchmarking models. SM stands for starting material

	Selection of the SM	Learning of Chemical Reactions	Generation of Synthesis Routes	Optimization Method
TRACER	*✓*	*✓*	*✓*	Monte Carlo tree search
Molecule Chef	×	*✓*	*✓*	Property predictor
DoG-Gen	×	*✓*	*✓*	Sampling and fine-tuning
CasVAE	×	×	*✓*	Bayesian optimization
SynFlowNet	×	×	*✓*	GFlowNet

#### Molecule chef

In the Molecule Chef, the reactants involving in chemical reactions are vectorized via graph neural networks (GNNs). A network for mapping to a latent space is learned by utilizing a “bag of reactants”, which is obtained by summing these vectorized representations. In the original paper, a property predictor was connected to the latent space, and the coordinates were updated by calculating the gradient of the latent variable to generate optimized molecules. For comparative experiments, the property predictor was trained by precalculating the QSAR values of the training dataset used in the original paper, which was derived from USPTO dataset.

#### DoG-Gen

DoG-Gen is the only model among the benchmark models in this comparative study that is capable of explicitly learning chemical reactions and handling multistep transformations. The mapping to the latent space was learned by encoding reaction trees, constructed from the USPTO dataset, with a two-stage Gated Graph Neural Network (GGNN). In the benchmark experiments, following the methodology of the original paper, 30 rounds of operations were performed. Each round involved sampling 7000 reaction trees from DoG-Gen and conducting fine-tuning twice with the top 1500 molecules ranked by their QSAR values. The same training dataset as that employed in the original paper, which was derived from USPTO, was used in this experiment.

#### CasVAE

In CasVAE, separate latent spaces are constructed for compounds and reaction trees, utilizing the pathways obtained by decomposing the molecules in the USPTO dataset with a reaction template-based retrosynthesis model^62^. Decoding is performed by using the latent variables sampled from each space. As per the original implementation, five batches of 3000 molecules were generated by applying Bayesian optimization to the latent space of the molecules.

#### SynFlowNet

SynFlowNet is a GFlowNet-based model that was designed to sample data points with a probability proportional to a reward value. The tasks of predicting applicable reaction templates for compounds and reaction partners for bimolecular reaction templates are performed via neural networks, and the structure expansion procedure based on chemical reactions is modeled as a Markov process. The original paper utilized building blocks derived from Enamine building blocks^63^. In this comparative experiment, the training data were replaced with the USPTO dataset using the same preprocessing procedure as the original paper to standardize the conditions. The experimental conditions were set to be identical to those in the original implementation; the batch size during the generation step was 64, the temperature parameter was 32, and the number of training steps was 1000.

#### Experimental results

For all the benchmarked models, their compound generation effects were investigated via QSAR models of DRD2, AKT1, and CXCR4 (Table 6). For TRACER, the optimized beam widths for each starting material presented in Tables 2–4 were used in combination. As additional evaluation metrics beyond those used in Section 2.2, the proportion of unique generated compounds (uniqueness) and the Fréchet ChemNet distance (FCD)^64^ were calculated to evaluate the similarity between the generated compound set and the training dataset. Cases where the numbers of unique compounds were less than 5,000 were excluded because the FCD was shown to be unreliable in the original paper when the number of compounds was small, particularly when it was below this threshold.

**Table 6:** Comparison among the molecular generative models targeting each protein

Protein	Model	Total	Uniqueness (%)	Uniqueness to USPTO (%)	FCD	QSAR > 0.5	
						Num	Diversity
DRD2	TRACER	96043	**89.2**	**99.8**	14.3	16372	0.523
	(Sum of SMs **1**–**5**)					(17.0%)	
	Molecule Chef	218823	80.0	91.1	2.60	1963	0.490
						(0.90%)	
	DoG-Gen	153199	73.1	41.3	2.97	26780	0.425
						(23.9%)	
	CasVAE	615	85.7	91.5	-	**201**	**0.526**
						(**32.7**%)	
	SynFlowNet	58680	10.9	99.6	**17.0**	3	0.343
						(0.09%)	
AKT1	TRACER	40959	82.3	**99.9**	**12.2**	10424	0.564
	(Sum of SMs **6**–**10**)					(25.5%)	
	Molecule Chef	174959	**99.5**	95.2	3.13	827	0.534
						(0.47%)	
	DoG-Gen	164369	74.2	46.2	3.14	34199	**0.639**
						(28.0%)	
	CasVAE	830	79.8	71.8	-	**295**	0.396
						(**35.5**%)	
	SynFlowNet	61999	2.42	**99.9**	-	87	0.551
						(0.14%)	
CXCR4	TRACER	80280	92.4	**99.8**	**11.2**	10834	0.552
	(Sum of SMs **11**–**15**)					(13.5%)	
	Molecule Chef	151469	**99.3**	94.1	1.92	765	0.578
						(0.51%)	
	DoG-Gen	143602	82.6	44.6	4.42	**29204**	**0.603**
						(**24.6**%)	
	CasVAE	540	74.3	48.1	-	63	0.476
						(11.7%)	
	SynFlowNet	62627	2.85	99.9	-	7	0.478
						(0.01%)	

In accordance with the original paper, which indicated that FCD calculations are unreliable when using fewer than 5000 compounds, FCD values are not shown for cases where the number of unique molecules was less than 5000.

The highest values or efficiencies are shown in bold.

When DRD2 was targeted, TRACER presented the greatest compound uniqueness and the largest proportion of generated USPTO-unique molecules. SynFlowNet generated a substantial number of duplicate compounds, resulting in only 10.9% unique compounds. DoG-Gen exhibited a USPTO-unique compound ratio of 41.3%, indicating a large number of generated compounds overlapping with the training data due to its learning of a mapping from the reaction trees to the latent space. Furthermore, the FCD values of both the Molecule Chef and DoG-Gen were low, suggesting that methods that construct latent spaces via reaction trees tend to explore regions that are close to the training data. SynFlowNet exhibited the highest FCD, which can be attributed to its reaction template-based approach, where various reaction templates can be applied to the starting materials to increase the FCD. A high FCD of 14.3 was also observed for TRACER, suggesting its success in the challenging task of generating compounds that are dissimilar to the training data while explicitly learning chemical reactions. CasVAE showed the best results in terms of the proportion of compounds with QSAR values greater than 0.5 and the overall diversity. This is likely because CasVAE performs Bayesian optimization solely on the latent space of the compound structures, thus performing a simpler task that does not consider the latent representations of reaction trees. Consequently, as shown in Section 2.4.3, CasVAE attempted to generate 15,000 reaction trees but yielded only 615 valid trees. This was mainly due to the selection of incorrect reaction templates during the reaction tree generation process and the failure to construct complete reaction pathways, resulting in a lack of high-throughput capabilities.

When targeting AKT1 and CXCR4, TRACER presented the highest values for the USPTO-unique compound ratio and FCD. This result indicates that TRACER robustly explored a vast chemical space with respect to the reward function while considering real chemical reactions. With respect to the proportion of compounds exceeding the QSAR threshold, CasVAE presented the highest value for AKT1, whereas DoG-Gen presented the highest proportion for CXCR4. As described in Section 5.2, the numbers of active compounds for DRD2, AKT1, and CXCR4 were 9,913, 4,035, and 925, respectively. This suggests that the performance of CasVAE was significantly influenced by the number of known ligands, which made it difficult to discover optimized molecules for CXCR4. DoG-Gen exhibited the highest degree of diversity, which is presumably attributed to the absence of an initial compound selection phase, allowing for a greater degree of freedom when exploring the chemical space. TRACER attained moderate performance in terms of the proportions of compounds generated with high QSAR values (17.0% for DRD2, 25.5% for AKT1, and 13.5% for CXCR4) compared with those of DoG-Gen and CasVAE. Notably, this ability significantly depends on the initially selected compounds. To evaluate the optimization ability of our framework with respect to the reward function, compounds with low QSAR values were intentionally set as the starting compounds. However, the other methods do not have such strong constraints, allowing for a greater degree of flexibility during the search process.

The means and standard deviations of the SA scores and molecular weights for the compounds generated by each model are shown in Table 7. With respect to the SA scores, all the models exhibited comparable performance, except for SynFlowNet, which targeted CXCR4. Although the SA score includes a size penalty term^17^, which was designed to penalize larger molecules, TRACER generated compounds with higher molecular weights than those of the Molecule Chef and DoG-Gen while maintaining comparable SA scores.

**Table 7:** Comparison among the SA scores and molecular weights of different models

Protein	Model	SA Score		Molecular Weight	
		Mean	Std	Mean	Std
DRD2	TRACER	2.52	0.378	412	69.6
	Molecule Chef	2.67	0.741	335	163
	DoG-Gen	2.55	0.488	373	125
	CasVAE	2.75	0.663	465	162
	SynFlowNet	2.84	0.453	449	93.3
AKT1	TRACER	2.77	0.448	411	74.7
	Molecule Chef	2.71	0.741	382	184
	DoG-Gen	2.74	0.466	321	102
	CasVAE	2.81	0.423	414	90.9
	SynFlowNet	2.79	0.244	466	35.3
CXCR4	TRACER	2.45	0.419	420	74.0
	Molecule Chef	2.60	0.660	347	158
	DoG-Gen	2.35	0.507	313	97.3
	CasVAE	2.70	0.375	395	79.5
	SynFlowNet	3.41	0.207	416	110

### Structural optimization experiments involving the QSAR model starting from unseen molecules

**Fig. 9:**
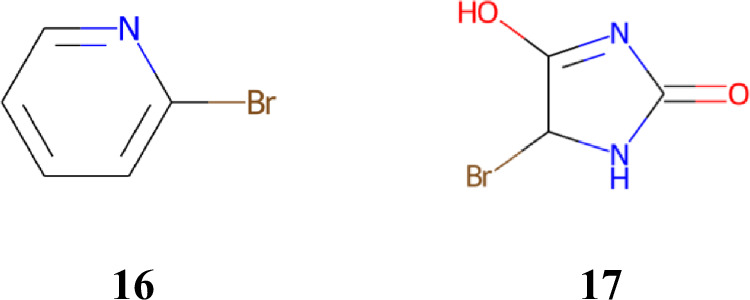
Set of molecules selected as starting materials (16 for DRD2 and AKT1, 17 for CXCR4). These compounds were selected from a dataset created by removing compounds used to train transformer and QSAR models from the ZINC dataset. For each target protein, compounds in the dataset were docked, and the one with the highest ligand efficiency was selected as the starting compound.

Structural optimization experiments designed to simulate the actual drug discovery process were conducted. A dataset consisting of nonpatented-like compounds was obtained from the ZINC database as described in Section 5.3. The compounds with the highest ligand efficiency (LE), which is defined as the absolute docking score normalized by the number of heavy atoms, were then selected as the starting materials for each protein. As a result, **16** was selected for DRD2 (docking score =  − 5.1, LE = 0.729) and AKT1 (docking score =  − 4.5, LE = 0.643), whereas **17** was selected for CXCR4 (docking score =  − 4.7, LE = 0.671) (Fig. 9).

The results of the experiments conducted using compounds **16** and **17** as starting materials are presented in Table 8. Although these initial compounds exhibited high LE, their QSAR values were 0.00, 0.01, and 0.01 for DRD2, AKT1, and CXCR4, respectively. However, TRACER enhanced the reward values by optimizing the structures of these compounds. Through a beam width investigation, compounds with QSAR values exceeding the threshold were generated at rates of 11.2% (beam width = 10) for DRD2, 24.5% (beam width = 10) for AKT1, and 9.81% (beam width = 50) for CXCR4. Consistent with the results presented in Section 2.2, the majority of the generated compounds were not included in the training dataset.

**Table 8:** Results of the experiment that started from unseen molecules in the ZINC dataset

Target	Starting Material (SM)	QSAR of the SM	Beam Width	Total	Uniqueness to USPTO (%)	QSAR > 0.5	
						Num	Diversity
DRD2	**16**	0.00	10	7542	98.6	**774**	0.510
						(**11.2**%)	
			20	14405	99.0	725	0.480
						(5.21%)	
			30	18362	99.1	503	0.436
						(2.80%)	
			40	25102	99.9	691	0.414
						(2.82%)	
			50	34404	99.0	865	0.419
						(2.58%)	
AKT1	**16**	0.01	10	5602	98.1	**1374**	0.600
						(**24.5**%)	
			20	13224	98.9	2861	0.604
						(21.6%)	
			30	17212	99.2	1756	0.588
						(10.2%)	
			40	20494	99.3	878	0.521
						(4.28%)	
			50	17722	99.2	1187	0.514
						(6.70%)	
CXCR4	**17**	0.01	10	4473	99.1	257	0.523
						(6.30%)	
			20	8081	99.5	600	0.531
						(8.05%)	
			30	12425	99.7	669	0.522
						(5.61%)	
			40	17525	99.8	1591	0.542
						(9.58%)	
			50	23491	99.9	**2226**	0.559
						(**9.81**%)	

The highest efficiency achieved for each beam width is shown in bold.

**Fig. 10:**
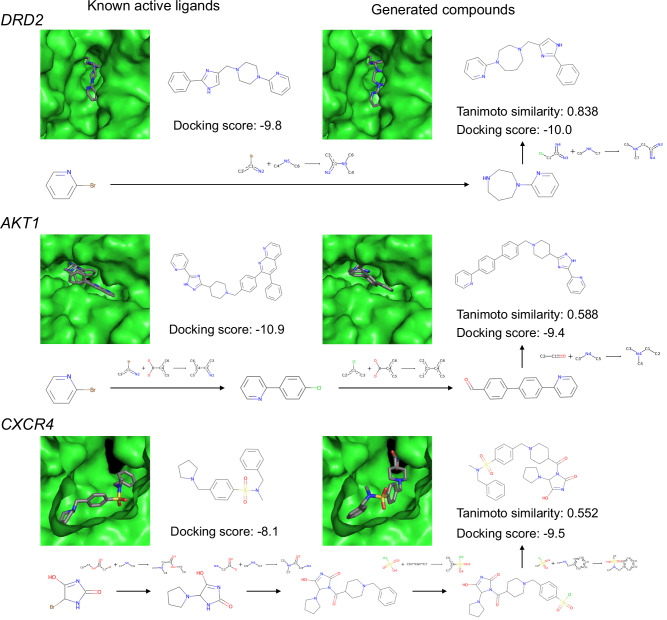
Structures and docking poses of the most similar compound pairs. The left column displays compounds found in known ligand datasets, and the right column shows compounds generated by TRACER. The figure also includes synthetic routes with reaction templates generated by our model, Tanimoto similarity scores for each compound pair, and docking scores.

For each target protein, the compound sets generated by TRACER were compared with known ligands. Pairwise Tanimoto coefficients were calculated between the known ligands and the compounds generated by TRACER with QSAR values greater than 0.5. The compound pairs with the highest similarity, the synthetic routes for the generated compounds, and the docking poses are shown in Fig. 10. Although these known ligands were not included in the training data, compounds with Tanimoto similarities of 0.838, 0.588, and 0.552 were generated for DRD2, AKT1, and CXCR4, respectively. For these generated compounds, TRACER generated synthetic routes consisting of 2, 3, and 4 steps. Although the QSAR model does not explicitly consider docking scores, these generated compounds exhibit comparable binding potential to active ligands. These results indicate that our framework can effectively generate optimized compounds for the reward function, even when starting from molecules that are not included in the training dataset. Furthermore, compounds that are structurally similar to active ligands that were not included in the training dataset could be generated via the QSAR model, along with by the generation of reaction pathways.

## Conclusions

In this study, a conditional transformer model was developed for the prediction of diverse chemical reactions. Compared with the unconditional model, the proposed model was shown to significantly improve the accuracy of the output product predictions. By incorporating reaction template information, the model was able to generate molecules through a wide variety of chemical reactions.

TRACER, which is a combination of a conditional transformer, a GCN, and an MCTS, was investigated for its ability to optimize compounds targeting specific proteins through the generation of synthetic pathways. MCTS-based molecule generation experiments using the QSAR model to predict the activity of DRD2, AKT1, and CXCR4 demonstrated the applicability of the conditional transformer in terms of drug discovery. MCTSs with various search widths showed that, in some cases, widening the search width enabled the efficient generation of compounds with high reward values. In contrast, in other cases, narrowing the search width facilitated a deeper search process, leading to the discovery of molecules with high reward values. Furthermore, the model performed structural transformations by appending commercially available building blocks, although it implicitly learned some of the reactants in the dataset. In benchmarking experiments, TRACER explored a chemical space farther from the training data than the other models did. Moreover, TRACER successfully generated compounds with high reward values, even when starting from compounds that were absent in the training data and with QSAR values close to zero. These results indicate the robustness of our framework against variations in the starting compounds and target proteins. The integration of additional models, such as a reaction condition recommendation model^65,66^ or a multiobjective optimization model^67^ for lead optimization, is expected to further accelerate the process of optimizing novel compounds in drug discovery tasks. Future work is needed to improve the robustness of the proposed approach by optimizing the model with larger datasets and exploring its potential applications involving diverse reward functions.

## Methods

**Fig. 11:**
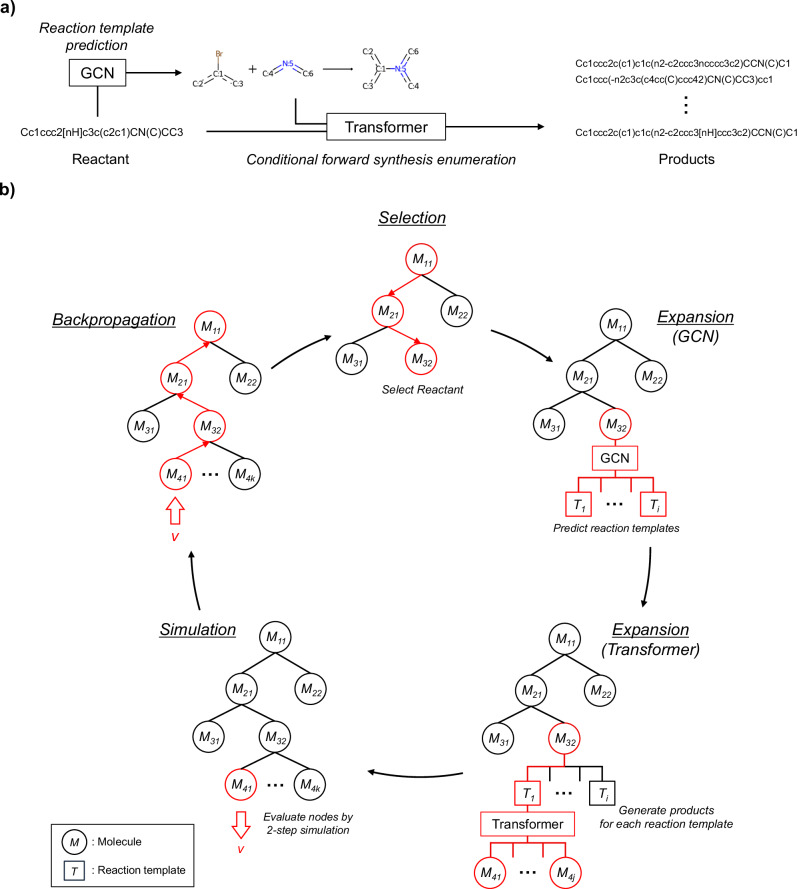
Comprehensive overview of an integrated model incorporating a conditional transformer and an MCTS. **a** Single-step forward synthesis enumeration. **b** An exploration process involving multistep synthesis pathway generation via an MCTS.

Figure 11a shows the procedure used by the proposed model to generate feasible products from the given reactant and to optimize the structure on the basis of virtual chemical reactions. First, a graph convolutional network (GCN) is used to predict the applicable reaction templates for the reactant. To filter mismatched templates, those with no substructures that match the reactant are removed at this time. Second, the transformer performs molecular generation under the conditions of the reaction type predicted for the reactant. This operation enables the model to be aware of how the reactant should be transformed into a product by learning real chemical reactions that correspond to the conditional templates. The number of outputs for each reaction type is examined in Section 2. The reaction templates with only one suitable product, such as the deprotection of nucleophilic substituents, are manually annotated with indices of the reaction templates. These templates are assumed to generate only a single molecule. Fig. 11b shows an overview of the optimization framework using an MCTS. In this study, the MCTS uses simulations to selectively grow a search tree and determine the most promising actions, in conjunction with the transformer and GCN. It searches for the optimal compounds through four steps: selection, expansion, simulation, and backpropagation. In the selection step, the most promising molecule is determined via the upper confidence bound (UCB) score described in Section 4.3. In the expansion step, the applicable reaction templates for the molecule are expanded, and the transformer performs conditional forward synthesis-based prediction for each reaction template. In the simulation step, the potential of the newly generated molecules is evaluated by further exploring the tree. Finally, the estimated values are propagated back to the root node, ensuring that molecules with higher values are more likely to be selected in the next cycle. The computing environment for all the experiments was an Ubuntu 22.04 OS, an Intel(R) Xeon(R) Gold 5318Y CPU, 128 GB of RAM, and an NVIDIA GeForce RTX 4090 GPU (with 24 GB of memory).

### Conditional transformer model

In recent years, the development of compound generation models based on transformer architectures^48^ has significantly advanced^68,69^. These models utilize attention mechanisms to learn long-range dependencies in sequential data such as SMILES strings. Notably, the Molecular Transformer^47^ achieved high accuracy in forward chemical reaction prediction tasks. It was trained on the USPTO dataset to predict the products from the given reactants. Unlike the previously developed forward synthesis prediction models that generate products when provided with all starting materials, our model was developed to predict compounds that can be synthesized from a single starting material through various chemical reactions.

**Fig. 12:**
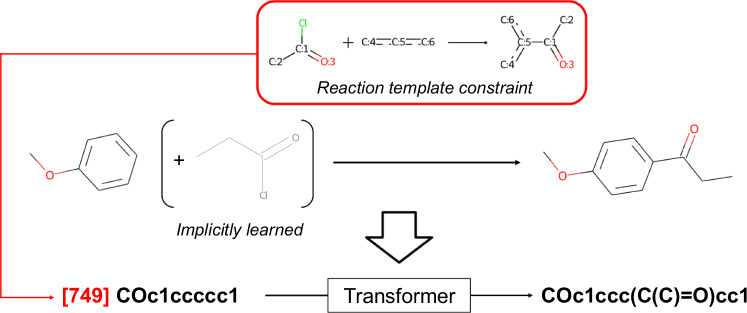
Procedure used by the conditional transformer to learn structural transformations under the conditions of the reaction template. The index of the reaction template is added to the beginning of the input SMILES sequence as a conditional token. In this example, the model implicitly learns the propionyl chloride from the information of the reaction template, the reactant, and the product.

The conditional transformer was employed to control the diversity of virtual chemical reactions. Previous studies on transformer models have reported that the properties of generated molecules can be successfully controlled by appending conditional tokens corresponding to the desired properties^70–72^. In this study, indices of the 1,000 reaction templates extracted from the USPTO 1k TPL dataset^73^ were added to the beginning of the input SMILES sequences, and the transformer was trained to predict the product corresponding to the reaction (Fig. 12). These manipulations were performed to control the diversity of the chemical reactions by conditioning various reaction templates on the reactants. As described in the Section 5.1, the reaction dataset considers only a single reactant as an explicit input. However, in most cases where partner compounds exist, they can be determined from the reaction template and the structural differences between the reactant and the product. This training approach enabled the model to learn all the necessary information for describing the chemical reactions while avoiding the need to select partner compounds from tens of thousands of potentially existing molecules. Consequently, the model is expected to generate plausible structural transformations in reference to real chemical reactions.

To clarify the effect of the conditional tokens, conditional and unconditional transformer models were implemented via PyTorch^74^ and torchtext libraries. The hyperparameters were determined according to the original transformer model^48^ as follows: the batch size was 128, the dimensionality of the model was 512, the dropout ratio was 0.1, the numbers of layers in the encoder and decoder were 6, the dimensionality of the feedforward network was 2048, the activation function was a rectified linear unit (ReLU), and the number of attention heads was 8.

### Graph convolutional network (GCN)

The GCN model^75^ was implemented on the basis of a previous study that predicted reaction templates from the SMILES representations of compounds^76^. Molecules with *n* nodes are represented as ${{{\mathcal{M}}}}\equiv (A,E,F)$, where *A* ∈ {0, 1}^*n*×*n*^ is the adjacency matrix, $F\in {{\mathbb{R}}}^{n\times d}$ is the matrix of the node features, and *E* ∈ {0, 1}^*n*×*n*×*T*^ is the edge tensor when *T* types of bonds are present. Using these representations and **A** = {**A**^(*t*)^∣*t* ∈ *T*}, the molecules can be represented as ${{{{\mathcal{M}}}}}^{{\prime} }\equiv ({{{\bf{A}}}},F)$, where ${({{{{\bf{A}}}}}^{(t)})}_{i,j}=1$ if a bond of type *t* is present between nodes *i* and *j*. The architecture of a GCN typically consists of convolution layers, dense layers, and aggregation layers.

#### Graph convolution layer

A graph convolution is computed as follows:

$${{{{\bf{X}}}}}^{(l+1)}=\sigma \left({\sum}_{t}{\tilde{{{{\bf{A}}}}}}^{(t)}{{{{\bf{X}}}}}^{(l)}{{{{\bf{W}}}}}_{t}^{(l)}\right)$$

where ${\tilde{{{{\bf{A}}}}}}^{(t)}$ is a normalized adjacency matrix for bond type *t*, **X**^(*l*)^ is the input matrix of the *l*th layer, and ${{{{\bf{W}}}}}_{t}^{(l)}$ is the parameter matrix for the *l*th layer with a bond type *t*.

#### Dense layer

After applying the graph convolution, the output **X**^(*l*+1)^ is typically passed through a dense layer, which is also known as a fully connected layer. The dense layer independently applies a linear transformation to the features of each node. The output **X**^(*l*+1)^ can be expressed as follows:

$${{{{\bf{X}}}}}^{(l+1)}={{{{\bf{X}}}}}^{(l)}{{{{\bf{W}}}}}^{(l)}$$

where **X**^(*l*)^ is the input matrix, and **W**^(*l*)^ is the weight matrix of the dense layer.

#### Aggregation layer

**Fig. 13:**
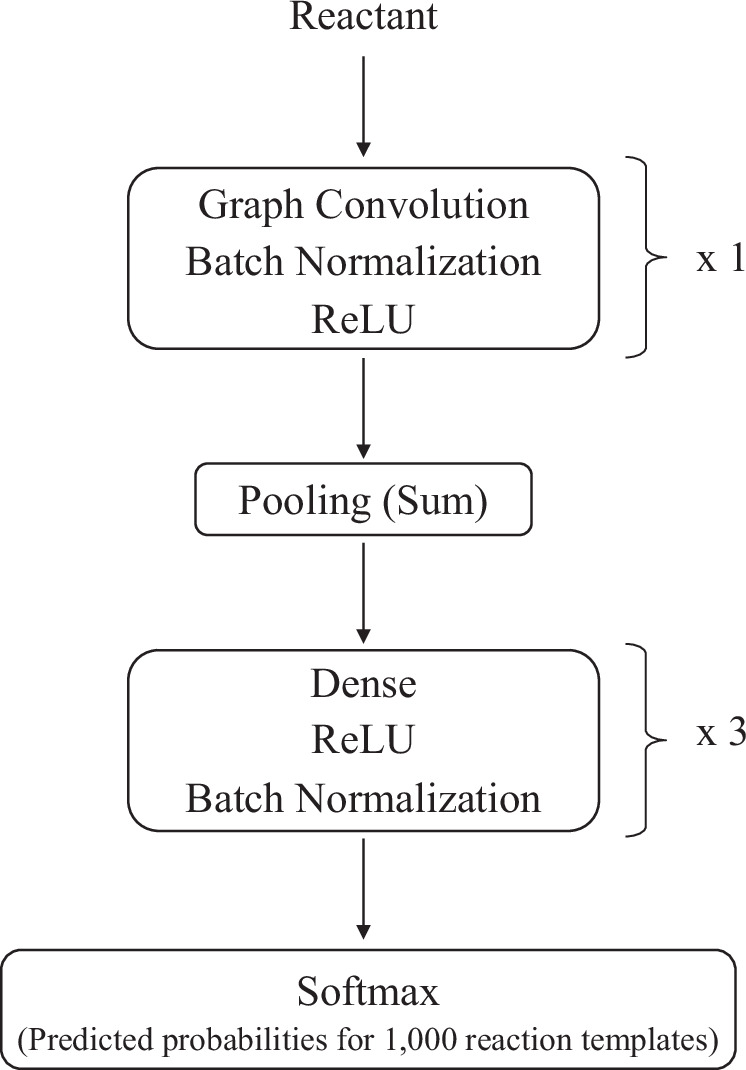
Architecture of the GCN model. The number of convolution layers and dense layers was determined using Optuna.The output dimension of the final layer was set to 1000 to predict reaction templates.

After the graph convolution and dense layers, the resulting node representations need to be aggregated to obtain a graph-level representation. Following previous work^76^, sum aggregation was employed. The output is calculated as follows:

$${\left({{{{\bf{X}}}}}^{(l+1)}\right)}_{j}={\sum}_{j}{\left({{{{\bf{X}}}}}^{(l)}\right)}_{ij}$$

where ${\left({{{{\bf{X}}}}}^{(l+1)}\right)}_{j}$ is the *j*th element of the vector representation. The graph-level representation **X**^(*l*+1)^ obtained through sum aggregation can be further processed to produce the final output of the GCN model. Additional dense layers are applied to predict the reaction templates. The overall architecture of the GCN model is shown in Fig. 13.

For the reactants included in the training data, the GCN model was trained to predict the correct reaction templates from 1,000 available templates. This implies that the random selection of a reaction template would result in an accuracy of 0.001. The hyperparameters of the GCN model were determined via Optuna^77^ (Table 9). Finally, the trained GCN model was evaluated on the test data, yielding a top-1 accuracy of 0.483, a top-5 accuracy of 0.771, and a top-10 accuracy of 0.862.

**Table 9:** The hyperparameter ranges in the GCN, where the values selected for the best model are shown in bold

Hyperparameters	Search range and best value
Dimension	128, **256**, 512
Convolution Layer	**1**, 2, 3, 4, 5
Dense Layer	1, 2, **3**, 4, 5
Learning rate	0.00001 – 0.1 **(0.0004)**

### Monte Carlo Tree Search (MCTS)

The MCTS algorithm^78^ was utilized to generate optimized compounds for the reward function. MCTSs integrated with RNNs, genetic algorithms, or matched molecular pairs^5–7,79–81^ have been demonstrated to be effective in molecular optimization tasks. In our framework, the nodes represent molecules, and the paths involving any nodes represent synthetic routes. The MCTS algorithm consists of four steps, each of which is detailed below.

1. Selection: Child nodes are iteratively selected by starting from the root node based on its estimated value and visit count. The tree policy, which is a score function for selecting child nodes, plays an important role in the performance of the MCTS. One commonly used metric is the UCB score, which was originally proposed for multiarm bandit problems. This approach balances the exploration of nodes with fewer visits and the exploitation of promising nodes. The UCB score for the node *i* is calculated via the following formula:

$$UCB={{{{\rm{arg\,max}}}}}_{i}\left\{Q({s}_{i})+2{C}_{p}\sqrt{\frac{\ln N({s}_{p})}{N({s}_{i})}}\right\}$$

where *Q*(*s*) represents the mean estimated value of state *s* for its child nodes and *N*(*s*) denotes the number of visits to state *s*. The states of each node *i* and its parent node are represented as *s*_*i*_ and *s*_*p*_, respectively. *C*_*p*_ balances the trade-off between exploitation and exploration. The first term in the UCB score corresponds to exploitation which encourages the selection of nodes with higher estimated values. The second term corresponds to exploration, which promotes the selection of the less-visited nodes. This selection policy guides the search toward the most valuable regions of the search space, enabling the discovery of the molecules that are likely to have the desired properties.

2. Expansion: The products generated via virtual chemical reactions using the transformer are added as new child nodes to the selected node. In the conditional transformer, the reaction templates that pass the substructure matching filter after sampling from the GCN are used as conditional tokens. In the unconditional transformer, the model simply predicts the products without considering the reaction templates. A beam search, which is a heuristic search algorithm that explores a graph by expanding the most promising nodes, is employed to obtain the feasible products, and the influence of the beam width on the molecular optimization process is investigated.
3. Simulation: The values of the child nodes generated in the expansion step are estimated. Virtual chemical reactions are repeatedly performed by the transformer a predetermined number of times. The number of reaction templates sampled during the simulation step is set to 5, and a single molecule is generated for each reaction template. The number of reaction steps explored is set to 2, and the maximum reward value among the generated compounds (25 or fewer) is set as the value for each node.
4. Backpropagation: The values calculated via the simulation step are propagated for all nodes along the path from the generated nodes to the root node, and the cumulative scores are calculated. The nodes in the direction of the promising compounds contained within the search tree are highly evaluated by this step; this step enables the efficient generation of compounds that are optimized for the evaluation function.

In this study, the quantitative structure-activity relationship (QSAR) model, which predicts the activity probability of a target protein from a chemical structure within the range from 0 to 1, was used as the reward function. The diversity of the generated compounds was evaluated via the following equation, as described in a previous benchmark study of molecular generative models^82^:

$${{\mbox{IntDiv}}}_{p}(G)=1-\root P \of{{\frac{1}{| G{| }^{2}}{\sum}_{{m}_{1},{m}_{2}\in G}T({m}_{1},{m}_{2})}}$$

where *G* is the set of valid compounds generated and *T* is the Tanimoto similarity between compounds *m*_1_ and *m*_2_ calculated by ECFP4 (2048-bit).

### QSAR model

The activity prediction models developed for dopamine receptor D2 (DRD2), AKT serine/threonine kinase 1 (AKT1), and C-X-C motif chemokine receptor 4 (CXCR4) were used as the reward functions for the MCTS. A random forest classifier was trained to predict the probability of an active compound on the basis of 2048-bit ECFP6 via active/inactive labeled datasets for each protein described in Section 5.2. The training dataset consisted of 9006 active and 306,457 inactive compounds for DRD2, 3623 active and 14,814 inactive compounds for AKT1, and 853 active and 3051 inactive compounds for CXCR4. The hyperparameters of these models were optimized via Optuna^77^ (Table 10). The performance of the trained model was evaluated by the area under the curve (AUC), which is a performance metric that evaluates the ability of a binary classification model to identify classes across a classification threshold, on a test dataset consisting of 907 active and 33,943 inactive compounds for DRD2, 412 active and 1,636 inactive compounds for AKT1, and 72 active and 355 inactive compounds for CXCR4. The models achieved an accuracy of 0.984 and an AUC of 0.703 for DRD2, an accuracy of 0.958 and an AUC of 0.897 for AKT1, and an accuracy of 0.974 and an AUC of 0.924.

**Table 10:** The ranges of the hyperparameters included in the random forest and the selected values for each target protein

Hyperparameters	Search range	DRD2	AKT1	CXCR4
bootstrap	True, False	False	False	False
max depth	1 – 500	429	410	498
max features	0 – 1.0	0.0749	0.0690	0.225
min sample split	2 – 5	2	2	5
min sample leaves	1 – 10	2	5	1
n estimators	1 – 300	151	158	223

### Selection of the starting compounds for the MCTS

The starting materials used by the MCTS for compound generation in Section 2.2 were randomly sampled from the distribution of the QSAR values for DRD2, AKT1, and CXCR4. Molecules with more than 8-membered rings and molecular weights ≥ 300 were removed from the USPTO 1k TPL dataset. Since reactants require functional groups that readily undergo chemical reactions, those not containing halogens, carbonyl groups, unsaturated bonds, or nucleophilic substituents were also removed; this decreased the number of compounds from 890,230 to 374,675. For each protein target, five initial compounds were selected as follows: the QSAR values of the filtered compounds were calculated, and one molecule was randomly sampled from each QSAR value bin including of 0–0.1, 0.1–0.2, 0.2–0.3, 0.3–0.4, and 0.4–0.5. These selected compounds are described in Section 2.2.

## Data

### USPTO dataset

In this study, the USPTO 1k TPL dataset reported by Schwaller et al. in their reaction classification work^73^ was used as a chemical reaction dataset. USPTO 1k TPL was derived from the USPTO database created by Lowe^83^ and consists of 445,000 reactions and corresponding reaction templates. The reaction templates were curated by selecting the 1000 most frequent template hashes obtained through atom mapping and template extraction via RxnMapper^84^. Certain reagents and solvents (e.g., hydrochloric acid, ethyl acetate, and dichloromethane) were removed from the dataset to further preprocess the data. The molecular pairs with the highest Tanimoto similarity between the reactants and products of each reaction were subsequently formed, indicating a structural transformation from the reactants to the products. The index of the labeled reaction template for each data point was appended to the beginning of the SMILES string of the reactants. The source code of TRACER, the activity prediction model, and the curated dataset are available in our public repository at https://github.com/sekijima-lab/TRACER.

### Dataset for QSAR modeling

The dataset for the QSAR modeling process targeting DRD2 was extracted from ExCAPE^85^ and ChEMBL^86^. In addition to the active/inactive labeled dataset derived from ExCAPE, a set of compounds from ChEMBL was added as active compounds. On the basis of the literature^87^, the active compounds retrieved from ChEMBL underwent filtration with the following parameters: standard relation equal to “=”, pChEMBL  > = 6.0, and molecular weight  < 750. The AKT1 and CXCR4, active/inactive labeled datasets acquired from DUD-E^88^ were combined with active compounds from ChEMBL. The active compounds obtained from ChEMBL were preprocessed via the same filtering criteria as those applied to DRD2. The final DRD2 dataset consisted of 9913 active and 340,400 inactive compounds, the AKT1 dataset consisted of 4035 active and 16,450 inactive compounds, and the CXCR4 dataset consisted of 925 active and 3406 inactive compounds.

### Dataset and docking simulations for structural optimization starting from unseen molecules

To demonstrate the ability of our framework to generate optimized compounds when starting from unseen molecules, a dataset of molecules not included in the training set was created. This dataset was constructed by removing the compounds used for training the transformer and QSAR models from the building blocks that were available in the ZINC database^89,90^. After applying the same filtering process described in Section 4.5, docking simulations were performed via Vina-GPU 2.0^91^ with DRD2 (PDB ID: 6CM4), AKT1 (PDB ID: 3CQW), and CXCR4 (PDB ID: 3ODU). The compounds with the highest ligand efficiency (absolute docking score divided by the number of heavy atoms) were then selected as the starting materials for each protein.

## Supplementary information

Supplementary Information

## Data Availability

The activity prediction model and the curated dataset utilized in this study are available in our public repository at https://github.com/sekijima-lab/TRACER.

## Code availability

The source code and trained models for TRACER have been released under the MIT license and are available in our public repository at https://github.com/sekijimalab/TRACER.
