# Supplementary material for: Molecular optimization using a conditional transformer for reaction-aware compound exploration with reinforcement learning
Source: Commun Chem. 2025 Feb 8;8:40. doi: 10.1038/s42004-025-01437-x (PMC11807120; doi:10.1038/s42004-025-01437-x)
Supplement: Supplementary file 1 — Supplementary Information [file 42004_2025_1437_MOESM1_ESM.pdf]

Supporting Information for Molecular Optimization Using A Conditional Transformer for Reaction-Aware Compound Exploration with Reinforcement Learning

Shogo Nakamura[1], Nobuaki Yasuo[2], and Masakazu Sekijima[*, 3]

[1]Department of Life Science and Technology, Institute of Science Tokyo, Kanagawa, Japan
[2]Tokyo Tech Academy for Convergence of Materials and Informatics (TAC-MI), Institute of Science Tokyo , Meguro-ku, Tokyo, Japan
[3]School of Computing, Institute of Science Tokyo, Kanagawa, Japan
[*]Email: sekijima@comp.isct.ac.jp

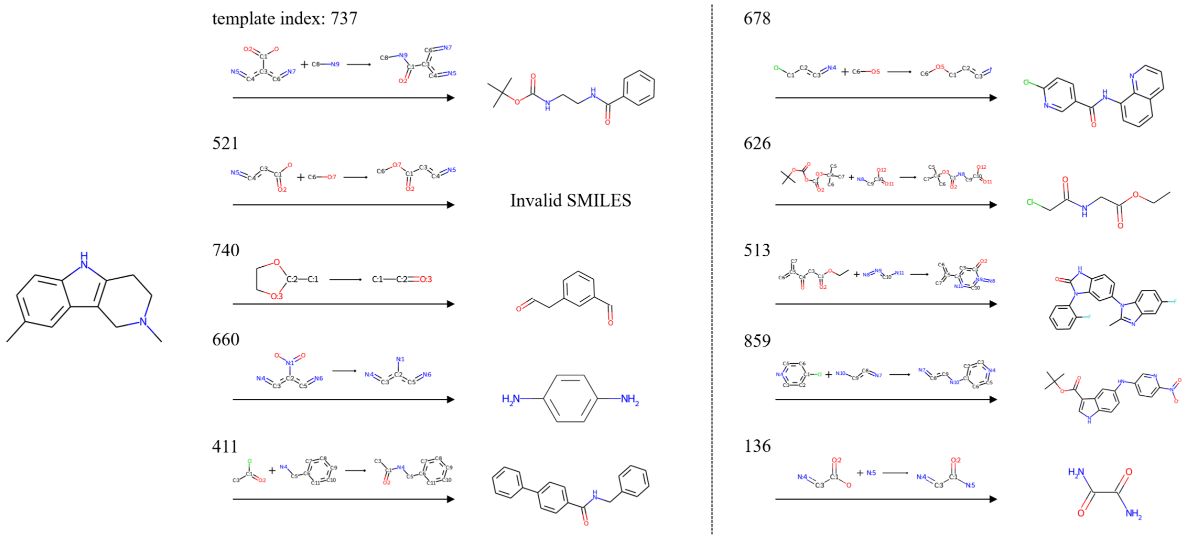

Supplementary Fig. 1. Examples of generated compounds conditioned with unmatched reaction templates.

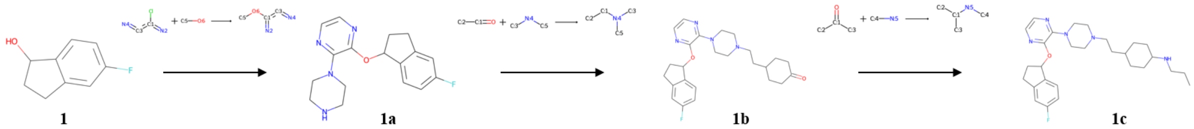

1 1a 1b 1c

reaction step	compound	CAS No.	number of suppliers	number of preparations
1	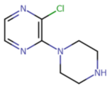 1-(3-chloro-2-pyrazinyl)piperazine	85386-99-8	68	2
2	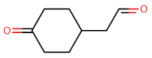 (4-oxocyclohexyl)acetaldehyde	606122-74-1	5	6
3	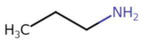 propylamine	107-10-8	52	211

Supplementary Fig. 2. Availability of reactants from Figure 9. The number of suppliers and preparation methods for each compound were searched on Reaxys on 2024/11/19.

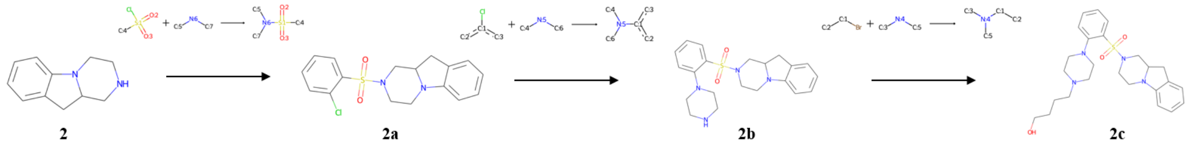

2 2a 2b 2c

reaction step	compound	CAS No.	number of suppliers	number of preparations
1	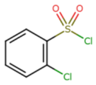 2-chlorophenylsulfonyl chloride	2905-23-9	87	13
2	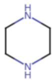 piperazine	110-85-0	78	252
3	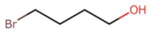 4-Bromo-1-butanol	33036-62-3	85	21

Supplementary Fig. 3. Availability of reactants from Figure 9. The number of suppliers and preparation methods for each compound were searched on Reaxys on 2024/11/19.

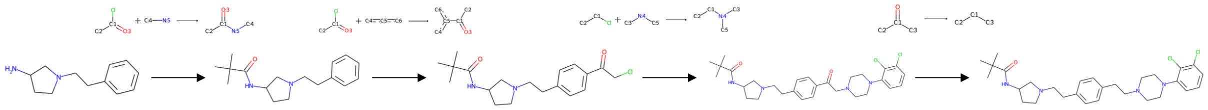

3 **3a** **3b** **3c** **3d**

reaction step	compound	CAS No.	number of suppliers	number of preparations
1	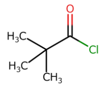 pivaloyl chloride	3282-30-2	41	34
2	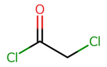 chloroacetyl chloride	79-04-9	49	63
3	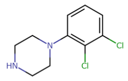 1-(2,3-dichloro-phenyl)-piperazine	41202-77-1	59	10

Supplementary Fig. 4. Availability of reactants from Figure 9. The number of suppliers and preparation methods for each compound were searched on Reaxys on 2024/11/19.

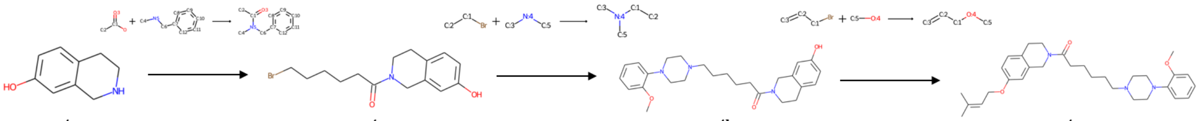

4 **4a** **4b** **4c**

reaction step	compound	CAS No.	number of suppliers	number of preparations
1	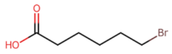 6-bromocaproic acid	4224-70-8	114	26
2	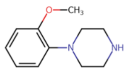 1-(2-Methoxyphenyl)piperazine	35386-24-4	84	28
3	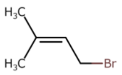 prenyl bromide	870-63-3	73	20

Supplementary Fig. 5. Availability of reactants from Figure 9. The number of suppliers and preparation methods for each compound were searched on Reaxys on 2024/11/19.

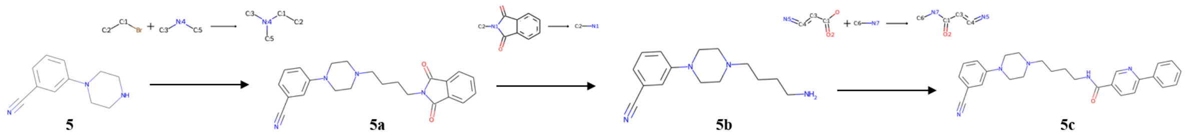

reaction step	compound	CAS No.	number of suppliers	number of preparations
1	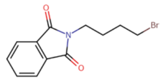 2-(4-bromobutyl)isoindoline-1,3-dione	5394-18-3	124	19
3	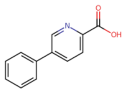 5-phenylpicolinic acid	75754-04-0	64	24

Supplementary Fig. 6. Availability of reactants from Figure 9. The number of suppliers and preparation methods for each compound were searched on Reaxys on 2024/11/19.

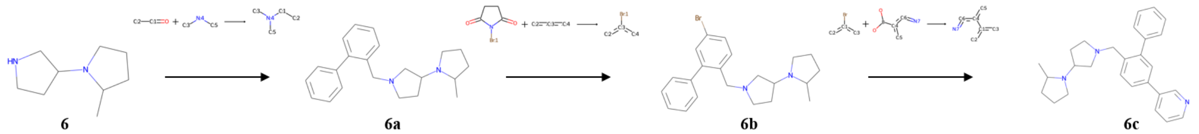

reaction step	compound	CAS No.	number of suppliers	number of preparations
1	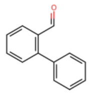 2-Phenylbenzaldehyde	1203-68-5	91	235
3	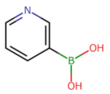 3-pyridylboronic acid	1692-25-7	123	17

Supplementary Fig. 7. Availability of reactants from Figure 10. The number of suppliers and preparation methods for each compound were searched on Reaxys on 2024/11/19.

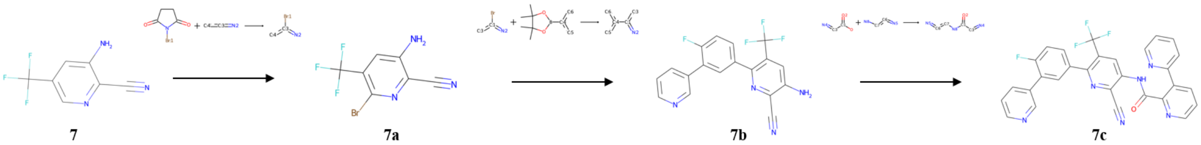

7 7a 7b 7c

reaction step	compound	CAS No.	number of suppliers	number of preparations
2	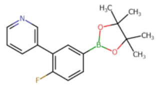 3-[2-fluoro-5-(4,4,5,5-tetramethyl-[1,3,2] dioxaborolane-2-yl)phenyl]pyridine	425378-79-6	7	7
3	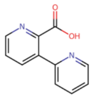 [2,3']bipyridyl-2'-carboxylic acid	1696775-28-6	21	1

Supplementary Fig. 8. Availability of reactants from Figure 10. The number of suppliers and preparation methods for each compound were searched on Reaxys on 2024/11/19.

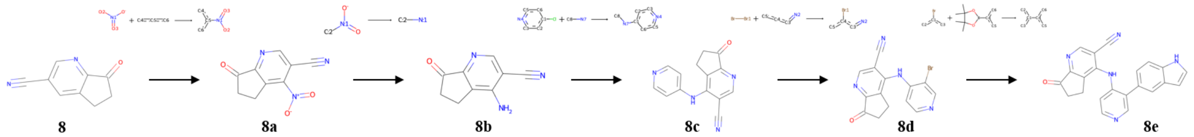

8 8a 8b 8c 8d 8e

reaction step	compound	CAS No.	number of suppliers	number of preparations
3	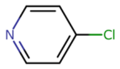 4-Chloropyridine	626-61-9	39	48
5	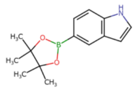 5-(4,4,5,5,-tetramethyl-1,3,2-dioxaborolan-2-yl)-1H-indole	269410-24-4	93	13

Supplementary Fig. 9. Availability of reactants from Figure 10. The number of suppliers and preparation methods for each compound were searched on Reaxys on 2024/11/19.

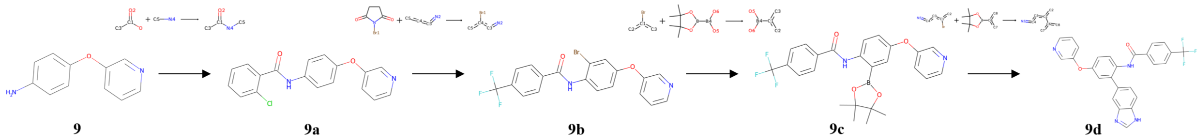

9 **9a** **9b** **9c** **9d**

reaction step	compound	CAS No.	number of suppliers	number of preparations
1	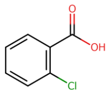 ortho-chlorobenzoic acid	118-91-2	99	218
3	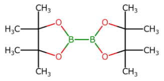 bis(pinacol)diborane	73183-34-3	126	44
4	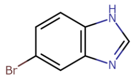 5-bromo-1H-benzo[d]imidazole	4887-88-1	81	24

Supplementary Fig. 10. Availability of reactants from Figure 10. The number of suppliers and preparation methods for each compound were searched on Reaxys on 2024/11/19.

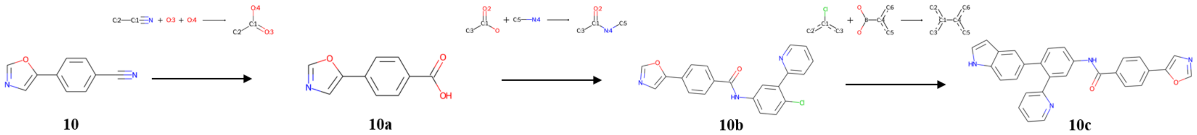

10 **10a** **10b** **10c**

reaction step	compound	CAS No.	number of suppliers	number of preparations
2	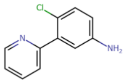 4-chloro-3-(pyridin-2-yl)aniline	879088-41-2	57	63
3	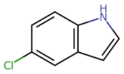 5-chloro-1H-indole	17422-32-1	138	51

Supplementary Fig. 11. Availability of reactants from Figure 10. The number of suppliers and preparation methods for each compound were searched on Reaxys on 2024/11/19.

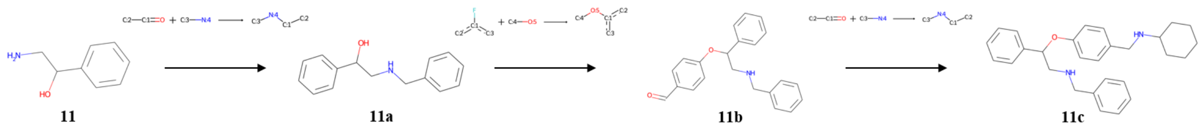

11 11a 11b 11c

reaction step	compound	CAS No.	number of suppliers	number of preparations
1	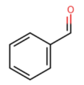 benzaldehyde	100-52-7	55	7,267
2	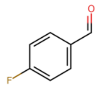 4-fluorobenzaldehyde	459-57-4	112	242
3	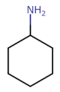 cyclohexylamine	108-91-8	67	450

Supplementary Fig. 12. Availability of reactants from Figure 11. The number of suppliers and preparation methods for each compound were searched on Reaxys on 2024/11/19.

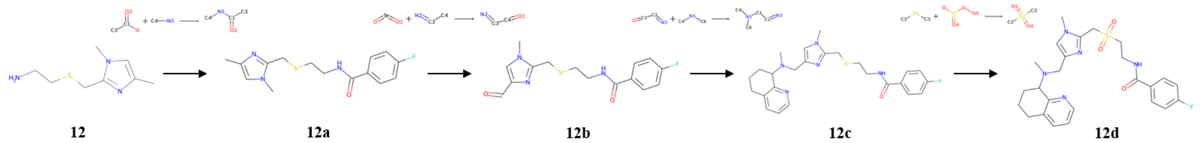

12 12a 12b 12c 12d

reaction step	compound	CAS No.	number of suppliers	number of preparations
1	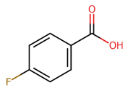 4-Fluorobenzoic acid	456-22-4	121	249
3	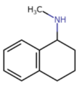 (-)-N-methyl-1,2,3,4-tetrahydro-1-naphthylamine	10409-15-1	46	6

Supplementary Fig. 13. Availability of reactants from Figure 11. The number of suppliers and preparation methods for each compound were searched on Reaxys on 2024/11/19.

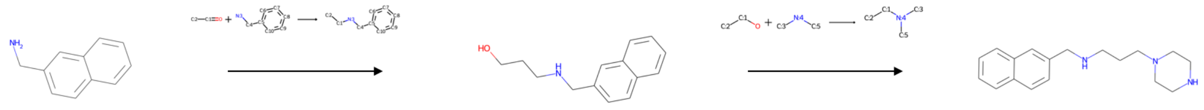

13 13a 13b

reaction step	compound	CAS No.	number of suppliers	number of preparations
1	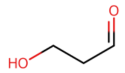 3-Hydroxypropanal	2134-29-4	49	46
2	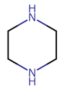 piperazine	110-85-0	78	252

Supplementary Fig. 14. Availability of reactants from Figure 11. The number of suppliers and preparation methods for each compound were searched on Reaxys on 2024/11/19.

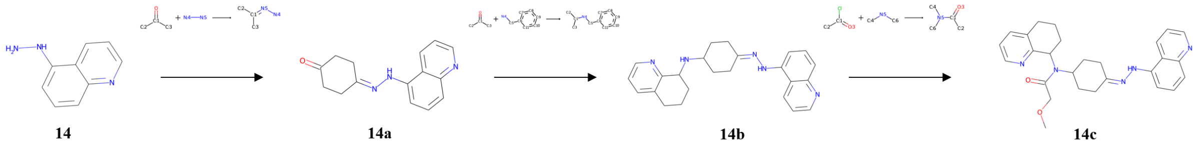

14 14a 14b 14c

reaction step	compound	CAS No.	number of suppliers	number of preparations
1	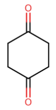 1,4-Cyclohexanedione	123-31-9	71	746
2	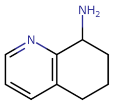 8-amino-5,6,7,8-tetrahydroquinoline	298181-83-6	95	11
3	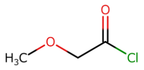 Methoxyacetyl chloride	38870-89-2	77	9

Supplementary Fig. 15. Availability of reactants from Figure 11. The number of suppliers and preparation methods for each compound were searched on Reaxys on 2024/11/19.

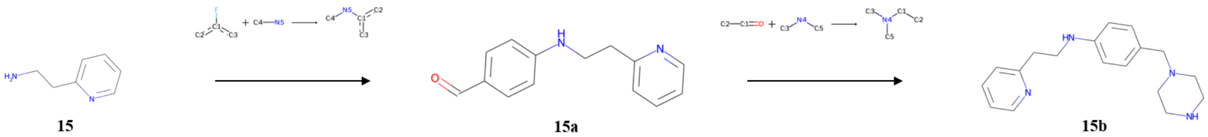

15 15a 15b

reaction step	compound	CAS No.	number of suppliers	number of preparations
1	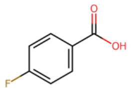 4-Fluorobenzoic acid	456-22-4	121	249
2	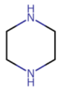 piperazine	110-85-0	78	252

Supplementary Fig. 16. Availability of reactants from Figure 11. The number of suppliers and preparation methods for each compound were searched on Reaxys on 2024/11/19.
